# Selective protection of human cardiomyocytes from anthracycline cardiotoxicity by small molecule inhibitors of MAP4K4

**DOI:** 10.1038/s41598-020-68907-1

**Published:** 2020-07-21

**Authors:** Pelin A. Golforoush, Priyanka Narasimhan, Patricia P. Chaves-Guerrero, Elsa Lawrence, Gary Newton, Robert Yan, Sian E. Harding, Trevor Perrior, Kathryn L. Chapman, Michael D. Schneider

**Affiliations:** 1 National Heart and Lung Institute, Imperial College London, London, W12 0NN UK; 2 Domainex Ltd, Chesterford Research Park, Little Chesterford, Saffron Walden, CB10 1XL Essex UK; 3 Present Address: The Hatter Institute, University College London, London, WC1E 6HX UK; 4 Present Address: Mechanistic Biology and Profiling, Discovery Sciences, R&D, AstraZeneca, Cambridge, CB4 0WG UK; 5 Present Address: Institute for Cancer Research, Sutton, SM2 5NG UK; 6 Present Address: The Francis Crick Institute, London, NW1 1AT UK

**Keywords:** Biotechnology, Cancer, Cell biology, Drug discovery, Stem cells

## Abstract

Given the poor track record to date of animal models for creating cardioprotective drugs, human pluripotent stem cell-derived cardiomyocytes (hPSC-CMs) have been proposed as a therapeutically relevant human platform to guide target validation and cardiac drug development. Mitogen-Activated Protein Kinase Kinase Kinase Kinase-4 (MAP4K4) is an “upstream” member of the MAPK superfamily that is implicated in human cardiac muscle cell death from oxidative stress, based on gene silencing and pharmacological inhibition in hPSC-CMs. A further role for MAP4K4 was proposed in heart muscle cell death triggered by cardiotoxic anti-cancer drugs, given its reported activation in failing human hearts with doxorubicin (DOX) cardiomyopathy, and its activation acutely by DOX in cultured cardiomyocytes. Here, we report successful protection from DOX in two independent hPSC-CM lines, using two potent, highly selective MAP4K4 inhibitors. The MAP4K4 inhibitors enhanced viability and reduced apoptosis at otherwise lethal concentrations of DOX, and preserved cardiomyocyte function, as measured by spontaneous calcium transients, at sub-maximal ones. Notably, in contrast, no intereference was seen in tumor cell killing, caspase activation, or mitochondrial membrane dissipation by DOX, in human cancer cell lines. Thus, MAP4K4 is a plausible, tractable, selective therapeutic target in DOX-induced human heart muscle cell death.

## Introduction

With earlier diagnosis and improved treatments for cancer, cardiovascular disease has become the main alternative cause of death in cancer survivors^1–4^. This heightened risk is ascribable to the cardiac toxicity of several routine anti-cancer agents including, especially, anthracyclines like doxorubicin (DOX)^5^. Though some relief can result from standard heart failure medications, no approved therapy apart from the iron chelator dexrazoxane addresses the responsible cytotoxic mechanisms and effector pathways operating in the damaged cardiomyocytes. For anthracyclines, these include diverse reported mediators—binding to nuclear topoisomerase 2β, thereby triggering DNA double-strand breaks, p53-dependent apoptosis, mitochondrial dysfunction, reactive oxygen species, and programmed iron-dependent cell death (ferroptosis)^1,6,7^. Cardiotoxicity can be acute, early, or late; early intervention based on subclinical abnormalities may be key to averting the delayed or cumulative effects^8^. Notably, acute toxicity is seen in up to 30% of patients receiving anthracyclines, soon after infusion^4^. Current cardioprotection trials in cancer rely chiefly on routine heart failure medications (ACE inhibitors, β-blockers, angiotensin receptor blockers), which mitigate the symptoms and signs, with little or no demonstrated impact on cardiomyocyte death. Therapeutic progress has been hampered, in part, by the failure of animal models alone to predict clinical success in cardioprotection. For instance, nearly all strategies for protection in ischemic heart disease, a more intensively studied indication, have failed between phases I and III, due to inadequate efficacy^9,10^. Conversely, this limitation has been compounded, historically, by the lack of workable systems for pre-clinical human validation. A further limitation, in the case of the cardiotoxicity of anti-cancer drugs, is the potential confounding effect of broadly cytoprotective therapies on the underlying therapeutic rationale for DOX, namely, tumour cell killing.

We recently developed novel, potent, highly selective, non-toxic inhibitors of Mitogen-Activated Protein Kinase Kinase Kinase Kinase-4 (MAP4K4), an “upstream” member of the MAPK superfamily that is a pivotal mediator of cell death in the heart^11^. MAP4K4 is activated in failing human hearts regardless of cause—including DOX cardiomyopathy—and in relevant rodent models including rat ventricular myocytes treated with DOX or more direct oxidative stress^11^. The causative role for MAP4K4 was shown by gain-of-function mutations in mice, by gain- and loss-of-function mutations in cultured rodent cardiomyocytes, and by gene silencing in human cardiomyocytes made from pluripotent stem cells (hPSC-CMs)^11^, an accessible, transformative human platform for cardiac target validation and compound development^12–18^. On the basis of these results, we then devised a small-molecule inhibitor of MAP4K4, designated DMX-5804, that rescues cell survival in hPSC-CMs subjected to simulated myocardial infarction^11^. The pathway for compound development progressed through an initial small compound screen against recombinant human MAP4K4, followed by 3D force field “pharmacophore” modeling (using inferred consensus features of the identified inhibitors as a virtual ligand to screen nearly 4 M structures in silico), testing the top 40 secondary hits empirically, and several years of structure-driven drug design to enhance potency, aqueous solubility, stability, and other pharmaceutical properties^11^. Exceptional selectivity was characteristic across the resulting chemical series, attributed to the very rare folded P-loop structure shared by the ATP-binding pocket of MAP4K4 with just a handful of other protein kinases^19^. Indeed, of more than 350 human protein kinases tested, only the two closest relatives, TNIK (MAP4K7) and MINK1 (MAP4K6), also were highly sensitive^11^.

Here, MAP4K4 inhibition has been subjected to rigorous target validation in human cardiomyocytes as a potential counter-measure against cardiotoxicity, conferring successful protection from DOX as measured by cardiomyocyte survival, suppression of apoptosis, and preservation of calcium cycling. Notably, under the conditions tested, protection was stringently selective for the human cardiomyocytes. No interference was seen in tumour cell killing by DOX, in any of five independent human lymphoma and myeloma cell lines.

## Results

### Inhibitors of MAP4K4 confer protection from DOX in rat H9c2 cardiomyocytes

**Figure 1:**
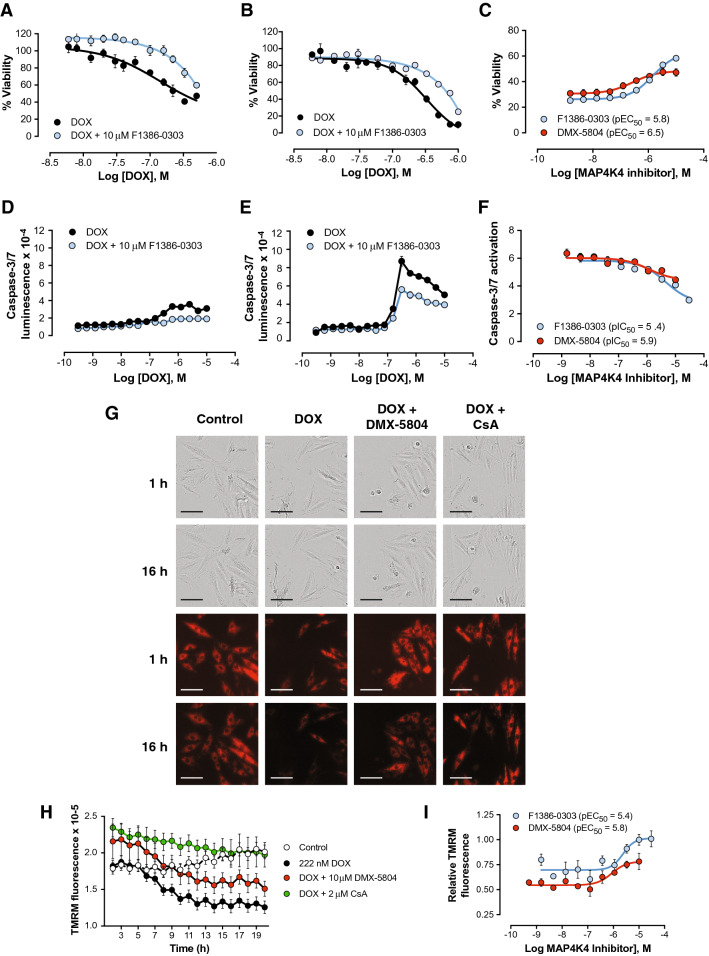
Inhibitors of MAP4K4 confer protection from DOX in rat H9c2 cardiomyocytes. H9c2 cells were treated with the MAP4K4 inhibitors shown, or DMSO as the vehicle control, beginning 1 h prior to DOX. (**A**–**C**) Viability, measured by the CellTiter-Glo assay. (**A,B**) Dose–response for DOX cardiotoxicity at 24 h (**A**) and 48 h (**B**), in the absence or presence of 10 μM F1386-0,303, the second-generation MAP4K4 inhibitor^11^. Data are triplicates, plotted as the mean ± SEM, and are representative of 3 independent dose–response experiments. (**C**) Protection from 333 nM DOX at 48 h by F1386-0303 versus DMX-5804, a third-generation inhibition^11^. Data are from a single experiment, plotted as the mean ± SEM, and are representative of ≥ 20 independent dose–response experiments across multiple batches of each compound. (**D**–**F**) Activation of executioner caspases. (**D,E**) Dose–response for caspase-3/7 activity triggered by DOX, at 6 h (**D**) and 24 h (**E**), in the absence or presence of 10 μM F1386-0303. Data are triplicates, shown as the mean ± SEM. (**F**) Dose–response for protection from 1 μM DOX at 24 h by F1386-0303 versus DMX-5804. Data are the mean ± SEM for quadruplicate cultures in three independent experiments. (**G**–**I**) Preservation of mitochondrial membrane potential (TMRM fluorescence). DOX, 222 nM; DMX-5804, 10 μM; CsA, 2 μM. (**G**) Representative photomicrographs at 1 and 16 h. Upper rows, phase-contrast microscopy of the fields shown below, for reference. Lower rows, TMRM fluorescence. Bar, 100 μM. (**H**) Time-course of TMRM fluorescence, measured hourly. Data are quadruplicates, plotted as the mean ± SEM, and are representative of 3 independent time-course experiments. (**I**) Dose–response comparing protection from 222 nM DOX at 16 h by F1386-0303 (pEC_50_ 5.9) versus DMX-5804 (pEC_50_ 5.5). Data are plotted as the mean ± SEM from 3 independent dose–response experiments run in triplicate.

As a starting point, initial proof-of-concept experiments were undertaken in rat H9c2 cardiomyocytes^20^, a low-cost, tractable model used most typically in toxicology research and early-stage cardiac drug discovery. Notably, this body of work includes more than 300 studies of anthracycline toxicity^21–34^ as well as high-throughput, phenotype-driven screens for novel cardioprotective agents^35,36^. Viability as measured by the CellTiter-Glo ATP generation assay was markedly impaired by DOX, with half-maximal effects at 150–250 nM at 24 and 48 h (Fig. 1A,B). The second-generation MAP4K4 inhibitor identified through field-point modeling, F1386-0303^11^, successfully interfered with H9c2 cell killing by DOX: 10 μM F1386-0303 reduced the cardiomyocytes’ sensitivity to DOX > threefold at both 24 and 48 h (pIC_50_ for DOX, < 6 versus 6.6; P < 0.05; Fig. 1A,B). In side-by-side dose–response comparisons, the more advanced, third-generation compound devised through structure-driven drug design, DMX-5804, was five-fold more potent than F1386-0303 in protecting DOX-treated cells (pEC_50,_ 6.5 versus 5.8; P < 0.001; Fig. 1C), consistent with its greater potency both against recombinant MAP4K4 and against cardiomyocyte death from oxidative stress^11^.

H9c2 cell protection by each of these MAP4K4 inhibitors was associated both with a block to activation of executioner caspases (Fig. 1D–F) and with preservation of mitochondrial membrane potential (Fig. 1G–I). A small increase in TMRM fluorescence over time was observed in control cardiomyocytes, ascribed to equilibration of the dye. Cyclosporine A (CsA) was used for comparison, given its known inhibition of the mitochondrial permeability transition pore, by targeting cyclophilin D^37^. Thus, small molecule inhibitors of MAP4K4 reduce DOX cardiotoxicity in this convenient non-human surrogate, acting at least in part through by preventing the dissipation of mitochondrial potential and cardiomyocyte apoptosis. These H9c2 data provided a direct justification for, and specifically guided, the experiments we next performed in hPSC-CMs.

### Inhibitors of MAP4K4 confer protection from DOX in human PSC-derived cardiomyocytes

**Figure 2:**
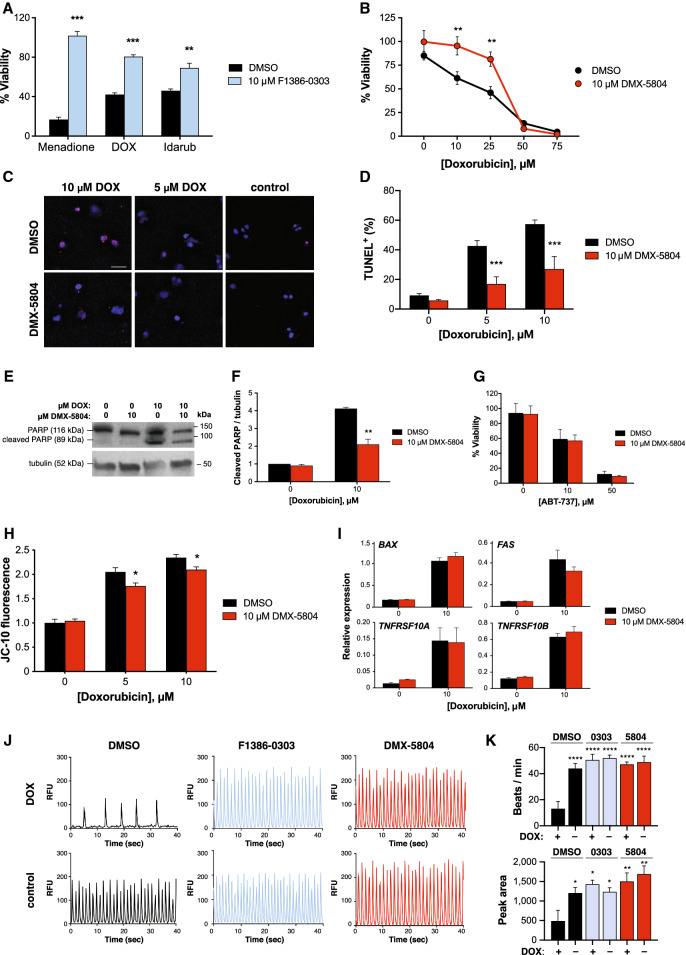
Inhibitors of MAP4K4 confer protection from DOX in human PSC-derived cardiomyocytes. (**A,B**) Protection of hPSC-CM viability (CellTiter-Glo). (**A**) Human vCorv.4U ventricular myocytes were treated for 72 h with 3 μM DOX, 2 μM Idarubicin, or 30 μM menadione, a proven MAP4K4-dependent death signal^11^, ± 10 μM F1386-0,303. Data were normalised to untreated and 0.1% Triton X-100-treated cells (100% and 0% viability, respectively). Data are triplicates, plotted as the mean ± SEM, and are representative of 3 independent experiments. **P ≤ 0.01; ***P ≤ 0.001. (**B**) Human IMR-90 cardiomyocytes were treated for 24 h with 0–75 μM DOX ± 10 μM DMX-5804. **P ≤ 0.01. (**C**–**F**) Suppression of hPSC-CM apoptosis. (**C,D**) Human vCor.4U cells were treated for 24 h with 0–10 μM DOX ± 10 μM DMX-5804 and were analyzed by TUNEL staining. (**C**) Representative images. Bar, 50 μm. (**D**) Data are the mean ± SEM of ≥ 3 replicates and are representative of 2 independent experiments. ***P ≤ 0.001. (**E,F**) IMR-90 cardiomyocytes, treated for 24 h as shown, were assayed by Western blotting for cleaved PARP1. (**E**) Representative Western blot (See also Supplementary Fig. S1). (**F**) Data are the mean ± SEM of 2 independent experiments. **P = 0.0016. (**G**) No protection from the Bcl-2 family inhibitor, ABT-737. IMR-90 cardiomyocytes were treated for 24 h as shown and analysed by the CellTiter-Glo viability assay. Data are the mean ± SEM of 4 replicates in each of two independent experiments. (**H**) Partial protection of ∆Ψ_m_. IMR-90 cardiomyocytes were treated for 24 h as shown and analysed by JC-10 fluorescence. Data are the mean ± SEM of 3 replicates in each of two independent experiments. *P ≤ 0.05. (**I**) No interference with DOX-induced expression of *BAX* or the indicated death domain receptor genes. IMR-90 cardiomyocytes were treated for 24 h as shown and analysed by by QRT-PCR. Data are the mean of 2 replicates in each of 2 independent experiments. (**J,K**) Preservation of Ca^2+^ cycling (FLIPR). vCor.4U cells were treated with sub-maximal (500 nM) DOX for 24 h, after 1 h pre-incubation with 10 μM F1386-0303 (blue) or DMX-5804 (red). Cardiomyocytes were monitored for 100 s and the first 40 s are illustrated. (**J**) Representative signals. RFU, relative fluorescence units. (**K**) Data are duplicates, plotted as the mean ± SEM from 3 independent experiments. By contrast to the loss of beat frequency and total peak area, only small changes occurred in median peak height and width (not shown). *P < 0.05; **P < 0.01; ****P ≤ 0.0001 versus DOX alone.

Despite the widely reported utility of H9c2 cells^21–36^, their applicability as a predictive model in cardiac drug development is compromised by their continuous proliferation, lack of the sodium/calcium exchanger NCX1, lack of beating, and skeletal muscle potential^20,38,39^. Moreover, explicit disparities have been reported in drug effects relating to cardioprotection and cardiotoxicity in this model, compared with human cells^40,41^. For this reason, we then tested the MAP4K4 inhibitors’ predicted ability to block cell killing by DOX in hPSC-CMs, a platform with greater fidelity to human cardiac biology and relevance to drug development^12–18^. Specifically, a functional requirement for MAP4K4 has been proven in this human model, in connection with other cardiac death signals^11^. In ESC-derived vCor.4U human ventricular myocytes, cell death induced by 3 μM DOX was reduced threefold by 10 μM F1386-0303, as measured by the CellTiter-Glo assay (viability increased from 42.2 ± 1.7% to 80.5 ± 1.9%; P < 0.001; Fig. 2A), with slightly less protection against a second anthracycline, Idarubicin (4-demethoxydaunorubicin; viability increased from 45.9 ± 1.8% to 69.1 ± 4.8%; P < 0.01). The sensitivity of this human model was comparable to the H9c2 cells used above, with half-maximal protection by F1386-0303 in both at a pEC_50_ of 5.8. In exploratory studies, similar protection of viability by the CellTitre-Glo assay likewise was shown in hiPSC-derived iCell cardiomyocytes, using 10 μM F1386-0303 and 2–20 μM DOX (data not shown). Analogously, protection was further confirmed in hiPSC-derived IMR-90 cardiomyocytes^42,43^ using DMX-5804, the third-generation MAP4K4 inhibitor^11^: e.g., cell viability in 25 μM DOX increased from 46.0 ± 6.4 to 81.3 ± 7.5% (P < 0.01; Fig. 2B).

As expected, apoptosis assessed by TUNEL staining and poly(ADP-ribose)polymerase-1 (PARP1) cleavage both were likewise inhibited (Fig. 2C–F). TUNEL staining for DNA fragmentation was reduced 2.5-fold, from 42.6 ± 3.7% to 16.9 ± 5.2% (tested in vCor.4U cardiomyocytes; P < 0.001; Fig. 2C,D) and cleaved PARP1 by 50% (tested in IMR90 cardiomyocytes; P = 0.0016; Fig. 2E,F). More detailed studies of apoptosis were then conducted in the IMR-90 cardiomyocytes. By contrast, DMX-5804 conferred no protection against a BH3-mimetic inhibitor of Bcl-2 and Bcl-xL, ABT-737 (Fig. 2G), which induces oligomerization of BAK and, hence, directly, mitochondrial pore formation leading to apoptosis^44^. This lack of protection suggests that DMX-5804 acts upstream from or in parallel with the dissipation of mitochondrial membrane potential (∆Ψ_m_), rather than at the level of downstream effectors. In agreement with this inference, ∆Ψ_m_ assessed by JC-10 fluorescence decreased in response to DOX and was partially protected by DMX-5804 (Fig. 2H). Therefore, DMX-5804 acts in part through preserving ∆Ψ_m_, albeit to a lesser extent than the observed effects on viability and apoptosis. An alternative mechanism proposed for DOX-induced cardiotoxicity is the up-regulation of death domain receptors, including FAS/TNFRSF6, DR4/TNFRSF10A, and DR5/TNFRSF10B^45^. However, no effect was seen on DOX-dependent expression of these genes in IMR-90 cardiomyocytes, measured 24 h after treatment (Fig. 2I).

A reported feature of human cardiomyocyte protection from acute oxidative stress by inhibitors of MAP4K4 was the preservation of spontaneous calcium oscillations^11^, a hallmark of cardiomyocyte function. Analogously, sub-maximal concentrations of DOX markedly impaired spontaneous calcium cycling in hPSC-CMs (beats min^−1^, 13.1 ± 5.5 versus 44 ± 3.8; P ≤ 0.0001), as seen in prior studies^12^, and this was fully rescued by co-administration of either MAP4K4 inhibitor, F1386-0303 or DMX-5804 (P ≤ 0.0001 for each; Fig. 2J,K).

### Inhibitors of MAP4K4 do not impair cancer cell killing by DOX

**Figure 3:**
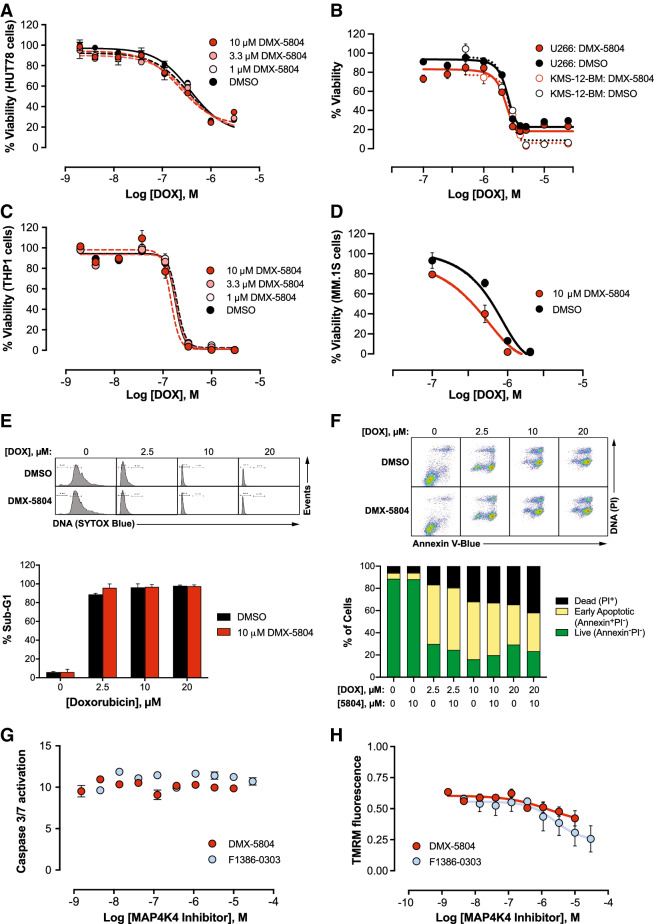
Inhibitors of MAP4K4 do not impair cancer cell killing by DOX. (**A**–**D**) Viability. Human tumor cells from the five indicated lines were treated with DOX and DMX-5804 at the concentrations shown and were assayed at 24 h (CellTiter Glo). Data are the mean ± SEM of ≥ 5 replicates and are representative of 2 independent dose–response experiments, excepting KMS-12-BM (5 replicates, 1 study). (**E**) Hypodiploid (sub-G1) DNA fragmentation. U266 cells were treated with 0–20 μM DOX for 24 h ± 10 μM DMX-5804, and DNA content was analysed by flow cytometry. Above, representative DNA histograms. Below, results are the mean ± SEM of single determinations in 2–3 independent experiments. (**F**) Annexin V. U266 cells were treated as in panel E then were analysed by flow cytometry for annexin V-Blue and PI fluorescence. Above, Representative scattergrams. Below, Data are single determinations, representative of two independent experiments. (**G**) Activation of caspase-3/7 (cf. Fig. 1E). THP1 cells were treated with 1 μM DOX for 24 h ± F1386-0303 at the concentrations shown. Data are the mean ± SEM of 4 replicates in each of 3 independent dose–response experiments, shown relative to the vehicle-treated control. (**H**) Loss of mitochondrial membrane potential. (cf. Fig. 1H). THP1 cells were treated with 1 μM DOX for 16 h ± F1386-0303 at the concentrations shown. TMRM fluorescence is shown as the mean ± SEM of 4 determinations in each of 3 independent dose–response experiments, relative to the vehicle-treated control.

Because a pro-survival agent, if non-selective in effect, might compromise the desired impact of DOX on killing cancer cells, a series of five human tumour lines was subjected to graded concentrations of DOX in the absence or presence of DMX-5804 (Fig. 3A–D). The tested cell lines were HUT-78 (T cell non-Hodgkin’s lymphoma), THP1 (acute monocytic leukemia), and U266, KMS-12-BM, and MM.1S (multiple myeloma). We chose to focus on these hemapoietic cancers in order to test multiple examples from a selected class, systematically, rather than canvas a wider array of cell types superficially. Second, we were guided by these specific lines’ known susceptibility to DOX^46–50^. Third, we chose to compare the three myeloma lines to test for potential differences in protection by our compound dependent on the status of p53 (U266 and KMS-12-BM, mutated; MM1.S, wild-type)^51^. No interference was seen in the DOX response, in any of these five human cancer lines. Analysis of hypodiploid DNA and annexin staining by flow cytometry further substantiated the lack of protection of cancer cells by DMX-5804 from DOX-induced cell death (Fig. 3E,F). Additional studies were performed, to monitor upstream events in the THP1 line (Fig. 3G,H). Executioner caspase activity was induced tenfold by 1 μM DOX, and was unaffected by either F1386-0303 or DMX-5804 (Fig. 3G). Likewise, the loss of mitochondrial membrane potential provoked by 1 μM DOX was unimpeded (Fig. 3H); rather, additional deterioration was seen, at the highest concentrations of both compounds. Thus, the inhibitors of MAP4K4 promote cardiomyocyte resistance to DOX without confounding effects on tumour cell death.

## Discussion

The unmet need for cardioprotection against the toxic effects of cancer chemotherapy poses a daunting challenge for the cardiologist and oncologist alike. As is true for cardiac muscle cell protection more generally, such as with acute ischemic injury, cardiac drug discovery has been slowed or stymied by the lack of human preclinical models for target validation and compound development^17,18^. The advent of hPSC-CMs provides an auspicious alternative to previous technologies, which has proven its predictive power at least in safety pharmacology^13,16^ and has justifiably raised expectations about its ability to distinguish or prioritise among potential remedies, in the years to come. Here, based upon the activation of endogenous MAP4K4 in DOX-induced cardiomyopathy and in cardiomyocytes treated acutely with DOX, we prove that DOX toxicity in two independent lines of hPSC-CMs is suppressed by two small-molecule inhibitors of MAP4K4. On the basis of these highly encouraging findings, we postulate MAP4K4 to be a well-posed target toward suppressing human cardiac cell death and dysfunction in drug-induced cardiomyopathies due to DOX and, perhaps, other chemotherapeutic agents*.* No agent for cardioprotection in cancer chemotherapy has ever entered human trials based on human preclinical proof of effect. Of course, future whole-animal studies in small and large mammals will be needed, to complement human cell-based evidence.

These findings are consistent with the proven benefits of blocking MAP4K4 to promote cell survival not only in hPSC-CMs subjected to alternative death signals (H_2_O_2_, menadione, hypoxia-reoxygenation)^11^, but also in adult mouse myocardium after experimental myocardial infarction^11^, human islet cells in palmitate-induced apoptosis^52^, and hPSC-derived motor neurons in amyotrophic lateral sclerosis^53^. Whereas this highly generalizable benefit speaks to the likely utility of MAP4K4 inhibitors beyond merely DOX-induced cardiotoxicity, such results also point to a reciprocal concern, that MAP4K4 inhibitors, if capable of acting as a broad survival signal, might interfere with tumour cell killing, notwithstanding current interest in the cancer field regarding the participation of MAP4K4 in tumor angiogenesis, tumor cell motility, and metastasis^54–59^.

A priori, the mechanistic basis for selective protection of cardiomyocytes but not tumor cells, strictly dichotomous across the seven human cell lines tested thus far, might relate to (i) DOX killing cardiomyocytes versus cancer cells in *distinguishable* ways, MAP4K4 inhibitors engaging preferentially the former, or (ii) DOX killing cardiomyocytes and cancer cells in *identical* ways, but with differing susceptibility to MAP4K4 inhibitors—in short, differences in the dying versus differences in the rescue, contingent on cell type. Provisionally, we favor the former of these overarching scenarios. At clinically relevant concentrations, the lethal effect of DOX in cancer cells is ascribed to interference with TOP2A, which is required during DNA replication, leading to DNA double-strand breaks^60^. By contrast, cardiomyocytes lack TOP2A, TOP2B largely mediates cardiotoxicity^61^, and greater emphasis is given instead to DOX-induced mitochondrial dysfunction and reactive oxygen species^7^. But, some overlap exists in these reported effector pathways, and the separation based on cell type is at present incomplete. Independently of TOP2A, DNA intercalation by DOX, DNA adduct formation, and activation of DNA damage responses by this alternative route can drive tumor cell death^62^, and DOX activates the DNA damage pathway in cardiomyocytes as well^63^. What can be said, thus far, is that protection from DOX occurred in both quiescent and proliferative cardiomyocytes (hPSC-CMs, versus H9c2 myoblasts), and therefore is unlikely due merely to the absence or presence of cell cycling, and that susceptibility to DOX remained in cancer lines, both in the absence and presence of wild-type p53, a gene known to mediate the cardiac toxicity^64^.

Resolving the molecular mechanism for cardioprotection by inhibitors of MAP4K4 will require identification of the responsible downstream MAP3Ks, terminal MAPKs, alternative downstream kinases, and non-canonical substrates, among other complementary strategies^53,55,56,65–69^. It is unknown whether MAP4K4 operates non-redundantly in driving human cardiac cell death from lethal chemotherapeutic drugs (our default hypothesis), equivalent to the unique role demonstrated by gene silencing in some other contexts: cardiomyocytes subjected to H_2_O_2_ as a model oxidative stress^11^ and motor neuron degeneration in amyotrophic lateral sclerosis^53^. The counter-hypothesis, redundancy with its closest relatives, MINK1 (MAP4K6) and TNIK (MAP4K7), which are identical in the ATP-binding pocket, is asserted for neuronal cell death after NGF withdrawal, based on combinatorial loss-of-function mutations^68^. A genetic dissection of DOX toxicity in hPSC-CMs could be instructive in distinguishing between these.

MAP4K4 has been implicated as a strong biomarker of cancer severity or prognosis, including evidence from histochemical levels^68,70^, gene expression^57,71^, microRNA networks^72^, and next-generation proteomics^73^. While the results shown here bear solely on cell survival, the demonstrated lack of a confounding effect on tumour cell killing by DOX gives obvious credence to further exploration of MAP4K4 inhibitors in these further aspects of cancer therapeutics, beyond just cardioprotection.

## Methods

### Cell lines

Two complimentary hPSC-CM lines were used, both with a proven role for MAP4K4 in cardiomyocyte death in other disease models^11^. Human vCor.4U PSC-derived ventricular myocytes were obtained from Ncardia. IMR90-4 hPSC-CMs were produced in-house using chemically defined medium, the Wnt inhibitors CHIR99021 and Wnt-C59, and metabolic selection in glucose-free medium^74^. Rat H9c2(2–1) cells^20^ (CRL-1446) were purchased from ATCC and cultured in Dulbecco's Modified Eagle's Medium (DMEM)-high glucose (D6546, Sigma-Aldrich) with 10% fetal bovine serum (FBS; F9665, Sigma-Aldrich) and 4 mM l-glutamine (G7513, Sigma-Aldrich). HUT-78T cell lymphoma (TIB-161) and THP1 acute monocytic leukemia cells (TIB-202) were obtained from the American Type Culture Collection. U266, KMS-12-BM, and MM.1S myeloma cells were kindly provided by Anastasios Karadimitris, Imperial College London.

### Compounds

The MAP4K4 inhibitors F1386-0303 and DMX-5804 were synthesized as previously reported^11^.

### Cell culture

Human vCor.4U cardiomyocytes were cultured on white (viability, CellTox Green) or black (FLIPR) clear-bottom 384-well plates (781098/781091, Greiner Bio-One), treated for 1 h with 50 μM fibronectin (Sigma-Aldrich), as described^11^. Thawed cells first were transferred from cryovials at 10,000 well^−1^ into 384-well plates containing pre-warmed Cor.4U maintenance medium for 4 days, with medium changes every 48 h. For viability and FLIPR experiments, cells were subjected to DOX (or comparator stress signals) ± test compounds in 40 µl well^−1^ of maintenance medium on day 4, and were assayed on day 5 or at other time-points as noted.

Human IMR90-derived cardiomyocytes were differentiated from the iPS(IMR90)-4 cell line (WiCell)^42^ by a modification of published methods^74,75^, as follows. Undifferentiated hiPSCs at 65–85% confluency were passaged using 0.5 mM EDTA and replated in E8 medium (Gibco) with 10 µM ROCK inhibitor (Y-27632; Selleckchem). At day 0 of differentiation, the medium was changed to RPMI supplemented with B27 without insulin (Thermo Fisher Scientific) and 6 µM CHIR99021 (LC Laboratories). On day 2, medium was changed to RPMI supplemented with B27 without insulin and the next day supplemented with 2.5 µM Wnt-C59 (Selleck Chemicals). On days 5, 7 and 9, the medium was replaced with fresh RPMI supplemented with B27 without insulin. For metabolic selection, the medium was replaced on days 11 and 13 with RPMI without D-glucose supplemented with B27, and then replaced on day 15 with RPMI supplemented with B27. The resulting cardiomyocytes were maintained for three weeks in serum-free RPMI-1640 (R8758, Sigma-Aldrich), supplemented with B27 and Antibiotic–Antimycotic (Thermo Fisher). The final concentration of amphotericin B was 270 nM, well below the threshold concentrations reported for toxicity of this compound^76,77^. The IMR90 cardiomyocytes were reseeded at a density of 12,500 cells well^−1^ onto half-area 96-well plates (675096, Grenier Bio-One), coated as above, for 1 week prior to treatment. The maintenance medium was replaced every 2 days, and treatments were performed in the maintenance medium.

H9c2 cells were provided at passage 2 from ATCC and were used only up through passage 16 or one month of culture. Cells were cultured to a maximum of 75% confluency before passaging in DMEM and 10% FBS. Cells were removed from the culture flask (typically, 75 cm^2^) using 0.25% (w/v) Trypsin-0.53 mM EDTA and passaged 1:2 every 3 days. Cells (1,000 well^−1^) were plated in white (viability, CellTox Green, caspase) or black (TMRM) clear-bottom 384-well plates (781098/781091, Greiner Bio-One) in DMEM and 10% FBS and were allowed to attach for 6–24 h before treatment.

THP-1 and HUT-78 cells were cultured with addition of fresh medium every 2–3 days. Cells were subcultured at a concentration of 1 × 10^6^ ml^−1^ with addition of media to a concentration of 3 × 10^5^ cells ml^−1^. Cells were used only up through passage 15 or one month of culture. The THP-1 and HUT-78 media comprised RPMI-1640 (30-2001, Sigma) with 10% or 20% FBS, respectively. Cells were seeded at 3 × 10^5^ ml^−1^ in 384-well clear-bottom plates as above, using RPMI-1640 and 10% FBS.

MM.1S, U266 and KMS-12-BM cells were cultured in RPMI‐1640 containing 10% FBS and Antibiotic–Antimycotic. Cells were seeded at a density of 25,000 cells well^−1^ in half-area 96-well plates (675096, Greiner Bio-One) and were serum-starved for 2 h before treatment.

Except where otherwise indicated, cells were pre-treated for 1 h with DMX-5804, then DOX (Sigma-Aldrich) was added for 24 h at the indicated concentrations. All cell lines were maintained in 5% CO_2_ and at 37 °C.

For in-cell dose–response experiments, MAP4K4 inhibitors were prepared in 96-well polypropylene plates as serial dilutions in DMSO of 10 mM stock solutions^11^. To a sterile 96-well intermediate plate, 1–3 µl of each well was added to 99 µl of medium, the samples were mixed, and 5 µl well^−1^ from the intermediate plate were transferred to the cells (final concentration, 0.1–0.3% DMSO; the final top concentration of inhibitor was, typically, 10–30 μM). Subsequently, DMX-5804 was used at 10 μM, added as 5 µl well^−1^ of a 100 μM stock solution. The death triggers including DOX were prepared fresh on the day of treatment at 10 × the final concentrations, and 5 µl well^−1^ were added as appropriate. Assay plates were incubated at 37 °C for the duration of treatment.

### Cell viability assays

#### ATP generation

Cell ATP levels were measured as described^11^. Assay plates were removed from the incubator and allowed to reach room temperature. CellTiter-Glo (CTG) reagent (Promega) was added (20 µl well^−1^), with gentle agitation for 30 min. Luminescence as a measurement of cellular ATP levels was read on a PHERAstar Plus microplate reader (BMG Labtech). Results were normalised to those of untreated control cells (no death signal, no MAP4K4 inhibitor) and to 100% cell death (addition of 0.1% Triton X-100, 2 h before CTG). Normalised values were plotted against the log concentration of the death inducer or inhibitor.

#### Caspase activity

The Caspase-Glo 3/7 Assay (Promega) was used to measure executioner caspase activity. Luminescence was measured 6–24 h after treatment (PHERAstar Plus) and is expressed as absolute luminscence or the ratio relative to vehicle-treated control cells.

#### Mitochondrial membrane potential (∆ψ_m_)

Tetramethylrhodamine, methyl ester (TMRM; Sigma-Aldrich) was added to cells at a final concentration of 30 nM, for 30 min in the incubator prior to treatment. TMRM fluorescence was measured using an IncuCyte S3 system (EssenBio; excitation 585 nm, emission 635 nm, 20 × objective) or CLARIOstar (BMG Labtech; excitation 544 nm, emission 645 nm, direct optic bottom reading). Using the IncuCyte, each plate was examined serially, every hour for 20 h. Data were determined as the integrated fluorescence intensity for 2 sites per well, using IncuCyte S3 software. The CLARIOstar readout was total fluorescence across the well, measured by spiral averaging. Data are shown as the integrated fluorescence (IncuCyte), total fluorescence (CLARIOstar), or ratio relative to vehicle-treated control cells.

Alternatively, ∆ψ_m_ was assessed in IMR-90 cardiomyocytes using the JC-10 Mitochondrial Membrane Potential Assay Kit—Microplate (Abcam). Cardiomyocytes were cultured on half-area, 96-well, black-walled, clear-bottom, fibronectin-coated plates (675096, Greiner Bio-One). JC-10 dye-loading solution was added at 25 µl well^−1^ and incubated in a CO_2_ incubator at 37 °C for 30 min followed by addition of 25 µl well^−1^ of assay buffer B. The fluorescence intensities of J-aggregates and monomeric forms were determined in a microplate reader using the excitation/emission wavelengths 490/525 nm and 540/590 nm, respectively. Data are expressed as the ratio of aggregate/monomeric forms, relative to untreated control cells.

#### TUNEL staining

vCor.4U cells were seeded at a density of 25,000 well^-1^ in full-area 96-well plates (Greiner Bio-One) and treated as detailed for the ATP viability assay. The cultures were then washed in cold PBS, fixed using 4% PFA for 15 min, and stained using the Click-iT Plus TUNEL Assay (Invitrogen). Fluorescence was scored using a Cellomics ArrayScan VTI High Content Screening platform and analysed using HCS Studio Software.

#### PARP1 cleavage

Cells were lysed in RIPA buffer (Cell Signaling Technology) with protease and phosphatase inhibitors (Cell Signaling Technology), resolved by SDS–polyacrylamide gel electrophoresis, and transferred to PVDF membranes (Bio-Rad) using the Trans-blot Transfer System (Bio-Rad) for Western blotting. Rabbit antibodies to PARP1 (1:1,000) and α-tubulin (1:1,000), and horseradish peroxidase-conjugated mouse anti-rabbit antibodies (1:10,000; all, Cell Signaling Technology) were used with enhanced chemiluminescence reagents (Pierce).

#### Flow cytometry

U266 and MM1.S cells were treated as above and fixed with 4% PFA for 15 min at 4 °C. Staining was performed using SYTOX Blue (Thermo Fisher) for hypodiploid DNA or the Pacific Blue Annexin V Apoptosis Detection Kit with PI (Biolegend), according to the respective manufacturer’s instructions. Flow cytometry was performed using an LSRII flow cytometer (Becton Dickinson) equipped with 355 nm ultraviolet, 405 nm violet, 488 nm blue, 561 nm yellow-green and 638 nm red lasers. Data were analysed using FlowJo v10, using the Cell Cycle plug-in.

### QRT-PCR

Gene expression was quantified as described^11^. RNA extraction was performed using PureLink RNA Mini Kits (Life Technologies). RNA quality and quantity were assessed using a NanoDrop 1000 spectrometer (Thermo Fisher Scientific). RNA was converted to cDNA using High-Capacity cDNA Reverse Transcription Kits (Applied Biosystems). QRT-PCR was performed using TaqMan Gene Expression Assays, MicroAmp Optical 384-well plates, 2X TaqMan Gene Expression Master Mix, and a QuantStudio G Flex Real-Time PCR System (Thermo Fisher Scientific). Results were normalized to *UBC* as the loading control.

### Calcium transients

Human cardiac calcium oscillation was assessed using FLIPR Tetra instrumentation and FLIPR calcium assay kits (Molecular Devices), as described^11^. vCor.4U cells were plated for 4 days, then were subjected to graded concentrations of DOX ± MAP4K4 inhibitors, as described for the viability assays. Cells were incubated for 24 h at 37 °C in 5% CO_2,_ using 25 μl well^−1^ of loading dye concentrate as the fluorescent calcium indicator (EarlyTox Cardiotoxicity Kit, R8210; Molecular Devices). Plates were then removed from the incubator and allowed to reach room temperature. Intracellular calcium oscillations were monitored using the excitation LED bank 470–495 nm and emission filter set 515–575 nm. Fluorescence intensity signals were acquired for 150 s at 2 ms intervals. Beat frequency, total peak area, median peak height, and median peak width were calculated and compared with the baseline control and with DOX alone. Peak parameters were evaluated using GraphPad Prism 6. Baseline fluorescence was removed for each well, and data were analysed as the area under the curve. Peaks were defined as ≥ 20% of the difference from minimum to maximum intensity.

## Quantitation and statistical analysis

Data are reported as the mean ± standard error, using a significance level of P < 0.05. Data were analyzed by one- or two-way ANOVA, using the Sidak or Bonferroni test for multiple comparisons and Welch’s t-test for pairwise comparisons (GraphPad Prism 7–8)^11^. Where multiple experiments are pooled, the technical replicates (separate cultures in each study) were averaged and treated as a single data point; where representative data are shown, indicative of additional independent studies, the replicates were analysed as separate data points. A 4-parameter fit was used to calculate pIC_50_ [− log IC_50_] and pEC_50_ [− log EC_50_] values.

## Supplementary information

Supplementary Information
