# Supplementary material for: Selective protection of human cardiomyocytes from anthracycline cardiotoxicity by small molecule inhibitors of MAP4K4
Source: Sci Rep. 2020 Jul 21;10:12060. doi: 10.1038/s41598-020-68907-1 (PMC7374628; doi:10.1038/s41598-020-68907-1)
Supplement: Supplementary file 1 — Supplementary Information [file 41598_2020_68907_MOESM1_ESM.docx]

Selective protection of human cardiomyocytes from anthracycline cardiotoxicity by small molecule inhibitors of MAP4K4

Pelin A. Golforoush^1,4^, Priyanka Narasimhan^2,5^, Patricia P. Chaves-Guerrero^1^, Elsa Lawrence^1^, Gary Newton^6^, Robert Yan^2^, Sian E. Harding^1^, Trevor Perrior^2^_,_ Kathryn L. Chapman^2,7^, and Michael D. Schneider^1,3^*

**Supplemental Information**

**Figure S1. Original, full-length Western blots used for Figure 2E.**


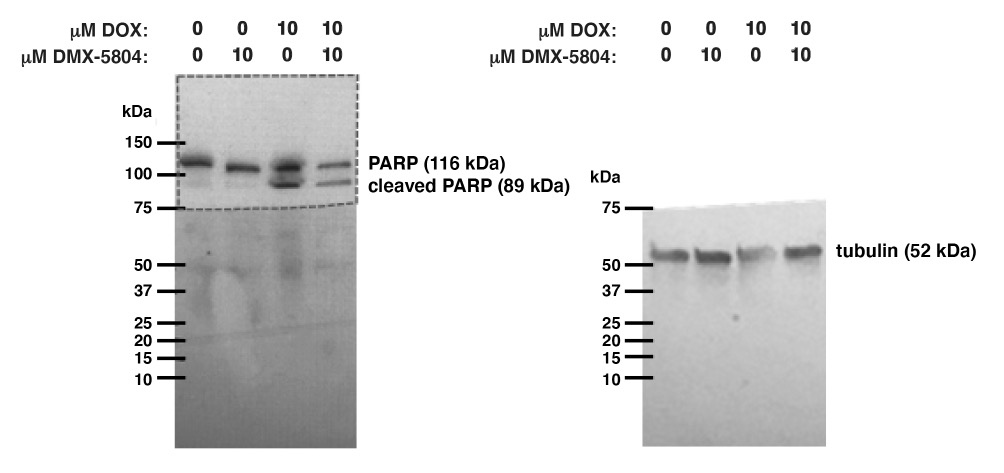


The original membrane was cut at 75 kDa and the upper section (highlighted by the dashed lines) was probed for cleaved and uncleaved PARP, and the lower section was probed unsuccessfully with a caspase-8 antibody. The lower section of the membrane was then stripped by incubation with agitation at room temperature for 10 min in 200 mM glycine, 0.1% SDS, 1% Tween-20, pH 2.2. The membrane was then washed for 10 min x 2 with PBS and for 5 min x 2 in 20 mM Tris, 150 mM NaCl, 0.1% Tween-20 (TBST). The membrane was then blocked for 1 hr in 5% milk in TBST, before being probed for tubulin.
